# Effect of dapagliflozin on proteomics and metabolomics of serum from patients with type 2 diabetes

**DOI:** 10.1186/s13098-023-01229-0

**Published:** 2023-12-04

**Authors:** Jia Liu, Xiaona Chang, Xiaoyu Ding, Xueqing He, Jiaxuan Wang, Guang Wang

**Affiliations:** Department of Endocrinology, Beijing Chao-Yang Hospital, Capital Medical University, NO. 8, Gongti South Road, Chaoyang District, 100020 Beijing China

**Keywords:** SGLT2i, Proteomics, Metabolomics, T2D

## Abstract

**Background:**

Sodium-glucose co-transporter 2 (SGLT2) inhibitors reduced the risk of cardiovascular and renal outcomes in patients with type 2 diabetes (T2D), but the underlying mechanism has not been well elucidated. The circulating levels of proteins and metabolites reflect the overall state of the human body. This study aimed to evaluate the effect of dapagliflozin on the proteome and metabolome in patients with newly diagnosed T2D.

**Methods:**

A total of 57 newly diagnosed T2D patients were enrolled, and received 12 weeks of dapagliflozin treatment (10 mg/d, AstraZeneca). Serum proteome and metabolome were investigated at the baseline and after dapagliflozin treatment.

**Results:**

Dapagliflozin significantly decreased HbA1c, BMI, and HOMA-IR in T2D patients (all *p* < 0.01). Multivariate models indicated clear separations of proteomics and metabolomics data between the baseline and after dapagliflozin treatment. A total of 38 differentially abundant proteins including 23 increased and 15 decreased proteins, and 35 differentially abundant metabolites including 17 increased and 18 decreased metabolites, were identified. In addition to influencing glucose metabolism (glycolysis/gluconeogenesis and pentose phosphate pathway), dapagliflozin significantly increased sex hormone-binding globulin, transferrin receptor protein 1, disintegrin, and metalloprotease-like decysin-1 and apolipoprotein A-IV levels, and decreased complement C3, fibronectin, afamin, attractin, xanthine, and uric acid levels.

**Conclusions:**

The circulating proteome and metabolome in newly diagnosed T2D patients were significantly changed after dapagliflozin treatment. These changes in proteins and metabolites might be associated with the beneficial effect of dapagliflozin on cardiovascular and renal outcomes.

**Supplementary Information:**

The online version contains supplementary material available at 10.1186/s13098-023-01229-0.

## Introduction

Type 2 diabetes (T2D) is a worldwide medical problem with increasing mortality and morbidity [1]. Individuals with T2D are more likely to develop coronary artery disease, heart failure, and chronic kidney disease [1]. Sodium‐glucose co‐transporter‐2 (SGLT2) inhibitors are a novel class of hypoglycemic agents that inhibit glucose reabsorption in the proximal tubules of the kidney [2–4]. Recently, Sodium‐glucose co‐transporter‐2 inhibitors (SGLT2i) have attracted tremendous attention because their cardiovascular and renal benefits are multifactorial and beyond glycemic control, but the underlying mechanism has not been well elucidated [2–4]. SGLT2 is a transmembrane protein almost exclusively expressed on proximal renal tubule [5]. Hence, it has been speculated that SGLT2i might exert beneficial effects by arousing a series of physiological consequences. Metabolomics and proteomics are emerging and powerful technologies in biological research [6]. Proteins are crucial effectors in the process of biogenesis, and their changes directly affect metabolic pathways in vivo [7]. Proteomics technology makes it possible to investigate the characteristics of proteins at a large-scale level, to obtain an overall and comprehensive understanding of the processes of disease occurrence, cell metabolism, growth and development, and various stress responses at the protein level [8]. Metabolomics is the collection of metabolites in a given biological sample [9]. Metabolites are usually downstream products of gene expression, transcription, and protein translation. In addition to genetic factors, metabolites may be affected by exogenous factors [9]. Since both proteins and metabolites are participants in metabolic pathways, influenced by nutrition, environment and physical condition, and reflect the overall state of human body, the combined multi-omics approach could provide insights into the complex interactions among different molecular pathways involved in SGLT2i in metabolic diseases. Importantly, T2D is a metabolic disease accompanied by the alteration of circulating proteins and metabolites [10, 11]. SGLT2i probably provide multi-organ improvement effect by affecting circulating proteins and metabolites. Considering the potential for protein and metabolite interactions, a multi-omics approach was employed for the study.

This study aimed to provide an integrated evaluation of the circulating proteome and metabolome in newly diagnosed T2D patients at the baseline and after dapagliflozin treatment.

## Methods

### Study design and participants

From April 2021 to August 2021, a total of 62 newly diagnosed T2D patients were consecutively enrolled at the Department of Endocrinology in Beijing Chao-yang Hospital Affiliated with Capital Medical University. All the patients had been diagnosed with T2D within the previous three months according to the 2020 American Diabetes Association diagnostic criteria, and met the following inclusion criteria: (1) aged 20–79 years old; (2) hemoglobin A1c (HbA1c) 7–10%. None of the patients had received anti-diabetic drugs before enrollment. None of the patients had any history of coronary artery disease, liver or renal function impairment, infectious disease, systemic inflammatory disease, hematological diseases, thyroid disease, autoimmune diseases, or cancer. Patients with ketoacidosis and hyperglycemic hyperosmolar status, and those who were pregnant or possibly pregnant, or ingesting agents influencing glucose or lipid metabolism were also excluded.

All the T2D patients received 12 weeks of dapagliflozin treatment (10 mg/d, AstraZeneca). During this time, the subjects received no other additional treatments. Patients were followed up every 4 weeks, and side effects were recorded at each visit. Three patients dropped out of the study due to urinary tract infections, and 2 patients dropped out due to self-discontinuation. During the 12 weeks of dapagliflozin treatment, hypoglycemia, ketoacidosis, or other side effects were not observed in any patients.

This study was conducted according to the principles of the Declaration of Helsinki, and approved by the Ethics Committee of Beijing Chao-yang Hospital Affiliated with Capital Medical University. Written informed consents were obtained from all participants.

### Clinical and biochemical measurements

The information about health status and medications was collected by two skilled nurses using a standard questionnaire. Clinical and biochemical measurements were performed at baseline and after 12 weeks of dapagliflozin treatment. Height and weight were measured to the nearest 0.1 cm and 0.1 kg by the same trained group, respectively. BMI was calculated as the weight in kilograms divided by the height in meters squared. Fasting venous blood was collected in the morning after an overnight fast. Biochemical parameters were measured immediately and serum was stored at − 80 °C for proteomic and metabolomic analysis after centrifugation at 1500*g* for 20 min at 4 °C.

Serum triglyceride (TG) was measured by a glycerol lipase oxidase reaction, total cholesterol (TC) by an enzymatic cholesterol oxidase reaction, and high-density lipoprotein cholesterol (HDL-C) and low-density lipoprotein cholesterol (LDL-C) by a direct assay using an autoanalyzer (Hitachi 747, Roche Diagnostics, Germany). Nonesterified fatty acid (NEFA) concentrations were determined by enzymatic colorimetric assays (Hitachi 747, Roche Diagnostics, Germany). Fasting blood glucose (FBG) was detected using the glucose oxidase method (Hitachi 747, Roche Diagnostics, Germany). Fasting plasma insulin (FINS) was determined by the chemiluminescence method (Dimension Vista, Siemens Healthcare Diagnostics, Germany). HbA1c was estimated by high-performance liquid chromatography using the HLC-723G7 analyzer (Tosoh Corporation, Tokyo, Japan). Serum creatinine (CREA) level was measured by the picric acid method (Hitachi 747, Roche Diagnostics, Germany). Homeostasis model assessment of insulin resistance (HOMA-IR) was calculated according to the following formula: HOMA-IR = FINS (mIU/L) × FBG (mmol/L)/22.5 [12]. The estimated glomerular filtration rate (eGFR) was calculated using the Chronic Kidney Disease Epidemiology Collaboration (CKD-EPI) equation [13].

### Proteomics

#### Chemicals and reagents

All chemicals used were mass spectrometry (MS) grade or higher. Methanol, acetonitrile (ACN), and formic acid (FA) were purchased from Fisher Scientific (Thermo Fisher Scientific, Inc. USA). Sodium dodecyl sulfate (SDS), ammonium bicarbonate (NH_4_HCO_3_), trifluoroacetic acid (TFA), DT-Dithiothreitol (DTT), iodoacetamide (IAA), lysyl endopeptidase (LysC), urea, and trypsin were obtained from Sigma-Aldrich (St. Louis, USA).

#### Sample preparation

Total protein concentration was measured by a BCA Protein Assay Kit (Thermo Fisher Scientific, Inc. USA). 10 μL aliquot of serum samples (~ 600 μg proteins) were transferred into High Select™ depletion spin columns to deplete the top 14 abundant blood proteins following the manufacturer’s instruction (Thermo Fisher Scientific, Inc. USA). Then, 320 μL samples were centrifugated at 12,000*g* for 10 min in a 10kD ultrafiltration tube (Millipore, Burlington, MA, USA). 200 μL of 8 M urea was added to the sample and centrifuged at 12,000*g* for 10 min twice, and finally, 200 μL of 8 M urea was added. DTT solution was added to the sample until reaching final a concentration of 10 mM, and incubated at 37 ℃ for 30 min. IAA solution was added to a final concentration of 20 mM, incubated away from light at room temperature for 30 min, and centrifugated at 12,000*g* for 10 min. 200 μL of 50 mM NH4HCO3 solution was added to the sample and centrifuged at 12,000*g* for 10 min twice, and finally, 200 μL of 50 mM NH4HCO3 solution was added. Next, 4 μg of LysC (50:1, w/w, protein: enzyme) was added to each sample for incubation at 37 ℃ for 2 h, after which 4 μg trypsin (50:1, w/w, protein: enzyme) was added to the sample for incubation at 37 ℃ overnight. Finally, a final concentration of 1% TFA was added to terminate the reaction. Next, 100 μL of methanol was centrifuged at 600*g* for 1 min in SoLAμ HRP plate (Thermo Fisher Scientific, Inc. USA), after which 100 μL of 80% ACN 0.1% TFA and 200 μL of 0.1% TFA were added for centrifuging at 1000*g* for 1 min, respectively. Then, the sample was loaded to the SoLAμ HRP plate for centrifuging at 1000*g* for 2 min twice. Next, 200 μL 0.1% TFA was added to the sample for centrifuging at 1000*g* for 2 min, after which 100 μL 80% ACN 0.1% TFA was added for centrifuging at 1000*g* for 3 min. Then the eluate was dried at 40 ℃ using a Centrifugal concentrator. Next, the digested and desalted peptides were dissolved to 0.5 μg/μL with 0.1% FA, after peptide assay by using NanoDrop microvolume spectrophotometer (Thermo Fisher Scientific, Inc. USA), and 2 μg was loaded into liquid chromatography-mass spectrometry (LC–MS) for data-independent acquisition (DIA) analysis.

#### LC–MS analysis

DIA proteomic analysis was performed by LC–MS analysis using a UltiMate™ 3000 RSLC nano-LC system (Thermo Fisher Scientific, Inc. USA) with Q Exactive HFX™ quadrupole-electrostatic field orbitrap high resolution mass spectrometry (Thermo Fisher Scientific, Inc. USA). XCalibur 4.3 (Thermo Fisher Scientific, Inc. USA) was used for data acquisition. All samples were allocated in a random order, and technicians were blinded to the status of the samples. Quality control (QC) samples (pooled samples from equal aliquots of each sample) were used to monitor the MS performance. Further details are provided in the Supporting Information section.

The digested peptide was loaded onto the Trap Column (Acclaim PepMap C18, 3 μm, 100 Å, 75 μm*2 cm, Thermo Fisher Scientific, Inc. USA) with buffer A (0.1% FA), and subsequently separated on the analytical column (Acclaim PepMap C18, 2 μm, 100 Å, 75 μm*25 cm, Thermo Fisher Scientific, Inc. USA). The trap column was eluted with different gradients of buffer B (0.1% FA, 80% ACN). The gradient of buffer B was from 3 to 6% in 3 min, 8% to 30% in 95 min, 30% to 99% in 4 min, and 99% to 99% in 5 min. The column flow rate was maintained at 300 nL/min.

A mass spectrometer with electrospray at an inlet voltage of 2.1 kV was used. The temperature of the heated capillary was set at 300 °C. After ionization, MS1 was performed using an Orbitrap Fusion Lumos (Thermo Fisher Scientific, Inc. USA). Fragmentation was achieved by high-energy collisional dissociation (HCD) with a collision energy of 32%. Data were obtained in DIA mode.

#### Data processing

DIA data were processed using DIA-NN 1.8 (The Francis Crick Institute, UK). A previously generated human chromatography library was used in the targeted analysis of DIA data against the human reference proteomics database. Default settings were used unless otherwise noted. Protein identifications were accepted if the false discovery rate (FDR) < 1% by the Scaffold Local FDR algorithm. When samples failed quality control, proteins were removed. Proteomic datasets were filtered for 75% valid values across all samples (proteins with > 25% missing values were excluded from downstream statistical analysis). Then, the K-nearest algorithm (sample-wise) was employed to impute the missing values. Protein intensities were then log-transformed for further statistical and bioinformatics analysis.

### metabolomics

#### Chemicals and reagents

All chemicals used were MS grade or higher. Methanol, ACN, and FA were purchased from Fisher Scientific (Thermo Fisher Scientific, Inc. USA). Ammonium acetate was purchased from Sigma-Aldrich (St. Louis, USA). Isotope labeling internal standards were purchased from Cambridge Isotope Laboratories (Tewksbury, MA, USA) and Toronto Research Chemicals (Toronto, Canada). Ultra-pure water (18.2 MΩ·cm) was prepared using a Milli-Q purified water system (Merck KGaA, Darmstadt, Germany).

#### Sample preparation

After thawing at 4 ℃, 120 μL of samples were transferred to a 96-well plate (Thermo Fisher Scientific, Inc. USA), and 480 μL methanol-ACN extract (containing isotope labeled internal standards: Taurine-d4 1.0 μg/mL, Hippuric acid-d5 1.0 μg/mL, Chlorophenylalanine 1.0 μg/mL, Acylcarnitine(12:0)-d9 0.2 μg/mL, Acylcarnitine(18:0)-d3 0.2 μg/mL, Palmitic acid-^13^C16 0.2 μg/mL, Stearic acid-d35 1.0 μg/mL, Chenodeoxycholic acid-d4 1.0 μg/mL) was added for vortex oscillation for 5 min. After centrifugation at 2000*g* for 20 min at 4 ℃, two 200 μL aliquots of each extract were transferred to another 96-well plate. The QC sample was prepared by mixing an equal aliquot of the supernatants from all samples. The extracts were concentrated and dried by decompressed centrifugation (Labconco Corporation, Kansas City, USA). After adding 80 μL polar complex solution, the supernatant of extracts was collected and transferred to a 96-well plate for further metabolomic analysis.

#### Ultra-high performance liquid chromatography-high resolution mass spectrometry analysis

The specific technical detection method was consistent with the study by Du et al. [14]. Non-targeted metabolomics analysis was conducted using a Ultimate™ 3000 ultra-high performance liquid chromatography coupled with Q Exactive™ quadrupole-Orbitrap high resolution mass spectrometer system (Thermo Fisher Scientific, Inc. USA). The hydrophilic fraction of metabolite extract was injected into the analytic workflow randomly. Technicians were blinded to the status of samples. Further details were provided in the Supporting Information section. All the data was acquired in profile format.

#### Data processing

Compound Discoverer software (Thermo Scientific, San Jose, USA) was used for comprehensive component extraction. The hydrophilic metabolites were structurally annotated by searching acquired MS2, local high-resolution MS/MS spectrum libraries, as well as mzCloud library (Thermo Scientific, San Jose, USA). Besides, the exact m/z of MS1 spectra was searched via a local HMDB metabolite chemical database. Mass accuracy of precursor within ± 5 ppm was the prerequisite, and a fit score of relative isotopic abundance pattern > 70% was employed to determine the chemical formula. Furthermore, retention time as well as high resolution MS/MS spectra similarity was employed to strictly confirm the structural annotation of metabolites. The area under curve value extracted by XCalibur Quan Browser information was used as the quantitative information of metabolites, and all peak areas data for the annotated metabolites were exported into Excel software for trim and organization before statistics. Finally, the chemical identification results were annotated with classification criteria proposed by MSI (metabolomics standardization initiative). The metabolomic data from the two measurements were merged and trimmed for further data process. MetaboAnalyst 4.0 (www.metaboanalyst.ca) was used to filter missing values by the following criterion: the metabolites with features > 50% missing values. The remaining missing values were replaced by half of the minimum positive value in the original data. The metabolomics data were then log-transformed for further statistical and bioinformatics analysis.

### Statistical and bioinformatical analysis

Differences in clinical parameters were analyzed using SPSS 22.0 (SPSS, Chicago, IL, USA). The distribution of continuous data was evaluated using Kolmogorov‐Smirnov test. For normally distributed data, continuous data were expressed as mean ± standard deviation. Because following the skewed distribution, the values of TG, FINS, and HOMA-IR were given as medians and upper and lower quartiles. Changes in parameters from baseline values within a group were evaluated using a paired *t*-test. Statistical significance was considered with two-tailed analyses as *p* < 0.05.

Principal component analysis (PCA) and orthogonal partial least square discriminant analysis (OPLS-DA) of proteins or metabolites were performed in SIMCA software (Umetrics AB, Umea, Sweden). A validation plot was used to assess the validity of the OPLS-DA model using permutation tests (n = 999). Differences of proteins or metabolites in baseline and after the treatment were analyzed by the Wilcoxon signed rank test for paired comparisons in the R statistical environment, version 4.1.3. Differentially abundant proteins or metabolites were identified by meeting the following criteria: (1) |log_2_ fold change (FC)|> 0.1375; and (2) the *p* value after the FDR multiple test correction (*q* value) < 0.05 by Benjamini–Hochberg method. Data visualization was conducted using R Studio [15, 16]. Gene Ontology (GO), Kyoto Encyclopedia of Genes and Genomes (KEGG) pathway analyses were conducted using the clusterProfiler package. Fisher’s exact test was used to evaluate the enrichment of the differentially abundant proteins, and a *p *value < 0.05 was considered significant. The correlations between differentially abundant proteins and metabolites were analyzed using Spearman rank correlation analysis, and heatmaps were drawn using R 4.1.3. The univariate receiver operating characteristic (ROC) curve by using the area under the curve (AUC) was applied to assess the accuracy of these changed metabolites and proteins to distinguish between the baseline and after dapagliflozin treatment.

## Results

### Influence of dapagliflozin on clinical parameters in newly diagnosed T2D patients

Metabolic parameters of the T2D patients before and after dapagliflozin treatment are summarized in Table 1. Dapagliflozin treatment significantly decreased FBG and HbA1c levels compared with baseline (both *p* < 0.01). BMI values were significantly decreased from baseline after 12 weeks of dapagliflozin treatment (*p* < 0.01). Compared with baseline, dapagliflozin treatment significantly decreased the FINS and HOMA-IR levels (FINS: *p* < 0.05; HOMA-IR: *p* < 0.01). However, the levels of TG, TC, HDL-C, LDL-C, NEFA, and CREA did not significantly change. Decreased eGFR values were also observed after dapagliflozin treatment (*p* < 0.05).

**Table 1:** Influence of dapagliflozin on clinical parameters in newly diagnosed T2D patients

Characteristic	Baseline	After treatment	*p value*
Age,y	47.5 ± 12.6		
Gender, F/M, n	28/29		
BMI, kg/m^2^	28.17 ± 2.70	26.57 ± 3.33	0.000
TG, mmol/L	1.75 (1.32–2.29)	1.48 (1.12–2.22)	0.083
TC, mmol/L	5.21 ± 1.04	5.10 ± 0.94	0.296
HDL-C, mmol/L	1.15 ± 0.27	1.18 ± 0.30	0.122
LDL-C, mmol/L	3.56 ± 1.02	3.43 ± 0.95	0.222
NEFA, mmol/L	0.71 ± 0.21	0.75 ± 0.22	0.199
FBG, mmol/L	8.78 ± 1.78	6.33 ± 0.91	0.000
FINS, mIU/L	11.60 (8.40–17.00)	9.60(6.65–14.92)	0.026
HOMA-IR	4.34 (2.97–6.43)	2.89 (1.75–4.14)	0.000
HbA1c, %	8.38 ± 1.05	6.59 ± 0.55	0.000
CREA, mmol/L	60.43 ± 11.66	61.77 ± 11.51	0.098
eGFR, mL/min/1.73m^2^	111.30 ± 10.51	109.91 ± 11.01	0.028

Data are mean ± standard deviation unless indicated otherwise. TG, FINS, and HOMA-IR are shown as medians, the upper and lower quartiles

T2D: type 2 diabetes; BMI: body mass index; TG: triglyceride; TC: total cholesterol; HDL-C: high-density lipoprotein cholesterol; LDL-C: low-density lipoprotein cholesterol; NEFA: Nonesterified fatty acid; FBG: fasting blood glucose; FINS: fasting insulin; HOMA-IR: homeostasis model assessment of insulin resistance; HbA1c: hemoglobin A1c; CREA: creatinine; eGFR: estimated glomerular filtration rate

### Proteomic analysis of the T2D patients at the baseline and after dapagliflozin treatment

In total, 1361 proteins were quantified from 114 samples (57 T2D patients at the baseline and after dapagliflozin treatment). After pre-processing and missing value filtering, 819 proteins were used for further analysis.

**Fig. 1:**
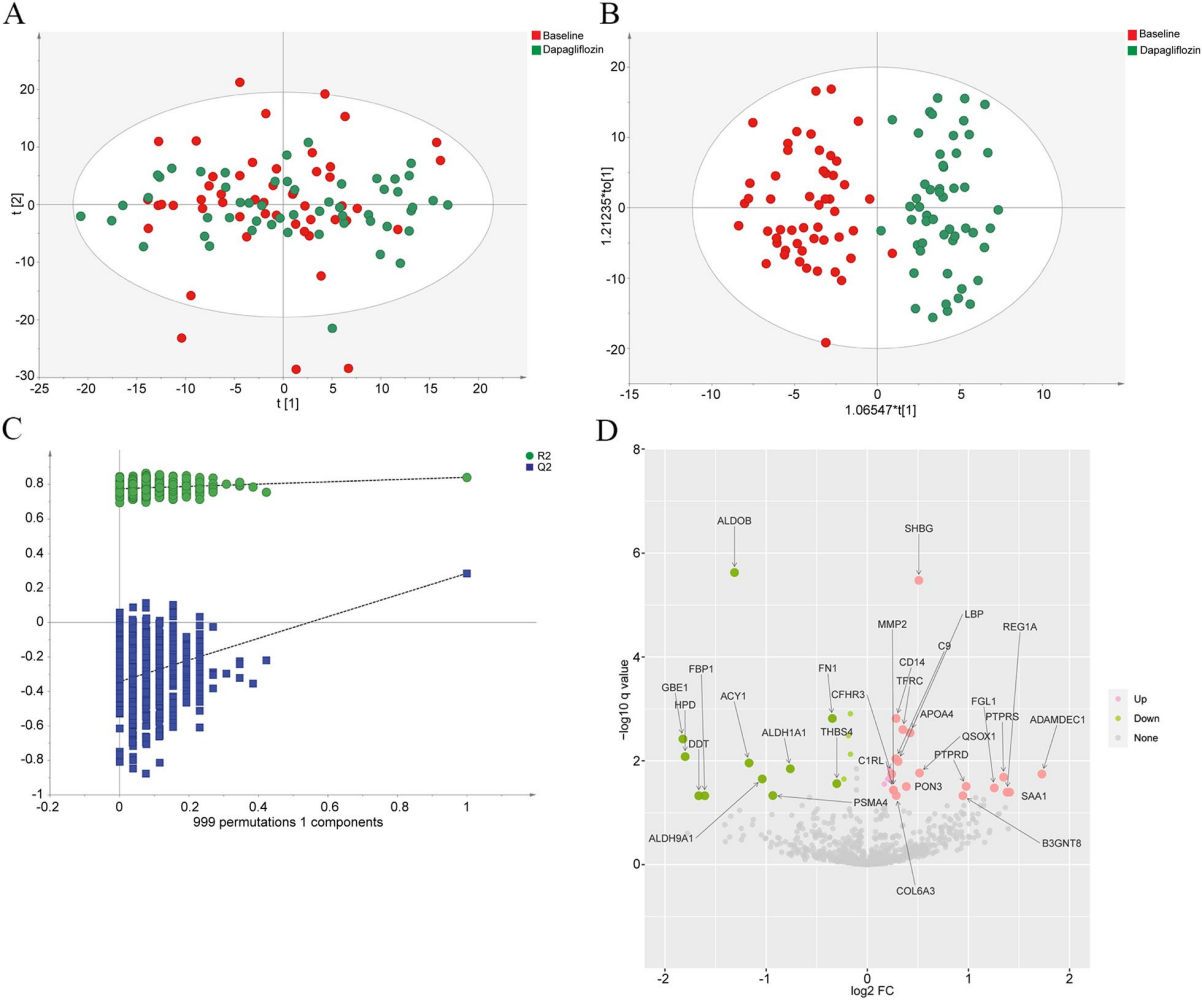
The differential protein expression analysis in the T2D patients at the baseline and after dapagliflozin treatment. **A** PCA score plot of proteomic data in the T2D patients at the baseline (red dots) and after dapagliflozin treatment (green dots). The cumulative fitness (R2 value) of the PCA model was 0.558. The t [1] and t [2] values in the figures represent the scores of each sample in principal components 1 and 2, respectively. Each dot on the plot represents a sample in the corresponding group. **B** OPLS-DA score plot of proteomic data in the T2D patients at the baseline (red dots) and after dapagliflozin treatment (green dots) (R2Y = 0.841, Q2 = 0.285). Each dot on the plot represents a sample in the corresponding group. **C** Permutation test of OPLS-DA (R2 intercept = 0.773, Q2 intercept = − 0.356). **D** Volcano plots of the differentially abundant proteins in the T2D patients after dapagliflozin treatment. The horizontal axis reflects fold change of the proteins after Log2 logarithmic conversion, and the vertical axis reflects FDR corrected p-value (q-value) after − Log10 logarithmic conversion. In the figure, the pink points indicate significantly differentially increased proteins, and green points indicate significantly differentially decreased proteins

First, principal component analysis (PCA) was used to assess the clustering of the T2D patients at the baseline and after dapagliflozin treatment (Fig. 1A). The OPLS-DA analysis indicated clear separations between the baseline and after dapagliflozin treatment in the T2D patients (R2Y = 0.841, Q2 = 0.285, Fig. 1B). The results of the permutation test strongly indicated that the original model was valid (R2 intercept = 0.773, Q2 intercept = − 0.356, Fig. 1C). A total of 38 proteins were identified, of which 23 were increased, and 15 were decreased (Additional file 8: Table S1 and Additional file 9: Table S2). All these identified proteins were further confirmed by the ROC curve analyses (AUC: 0.96, 95% CI 0.92–0.99, Additional file 1: Fig. S1). We also marked differentially abundant proteins on the volcano plots (Fig. 1D). Dapagliflozin treatment significantly increased sex hormone-binding globulin (SHBG), transferrin receptor protein 1 (TFRC), disintegrin and metalloprotease-like decysin-1 (ADAMDEC1), and apolipoprotein A-IV (APOA4) levels, and decreased fructose-1,6-bisphosphatase 1(FBP1), fructose-bisphosphate aldolase B (ALDOB), complement component C3, fibronectin (FN1), afamin (AFM), and attractin (ATRN) levels (Fig. 1D, Additional file 8: Table S1 and Additional file 9: Table S2).

### Functional enrichment analysis of differentially abundant proteins in the T2D patients after dapagliflozin treatment

**Fig. 2:**
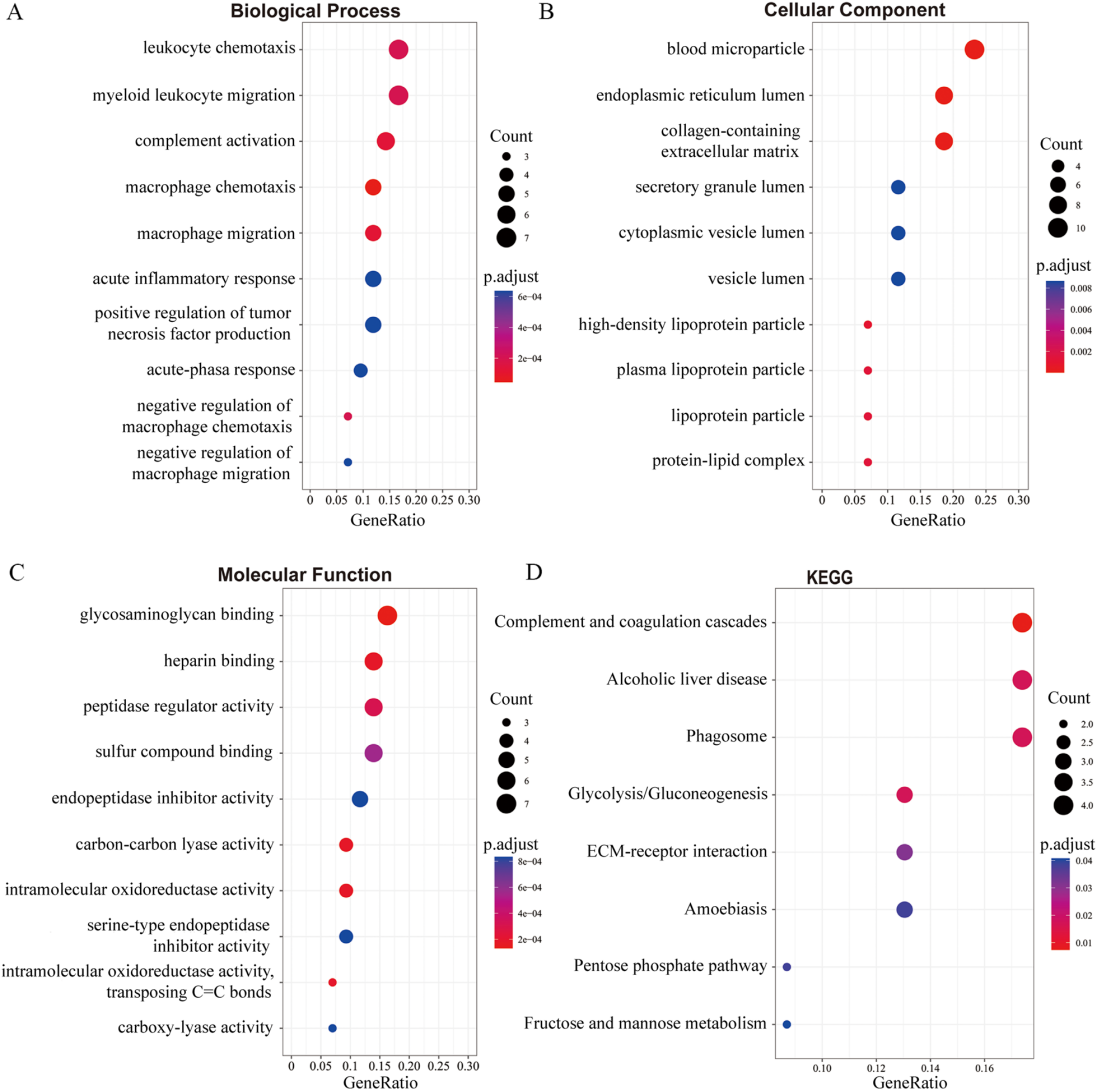
Functional annotation and enrichment analysis of the differentially abundant proteins in the T2D patients at the baseline and after dapagliflozin treatment. **A**–**C** GO functional enrichment analysis of the differentially abundant proteins in the T2D patients after dapagliflozin treatment. The three major categories of enriched GO functional classification: Biological Process (**A**), Cellular Component (**B**) and Molecular Function (**C**). **D** KEGG functional enrichment analysis of the differentially abundant proteins in the T2D patients after dapagliflozin treatment. The size of the dot represents the number of the differentially abundant proteins annotated to the pathways, and the color of the dot represents the FDR corrected p-value

To determine the characteristics of the differentially abundant proteins, we annotated the GO, and the KEGG pathway of the 38 differentially abundant proteins (Additional file 10: Table S3). The GO enrichment analysis showed that these differential proteins were mainly concentrated in leukocyte chemotaxis and myeloid leukocyte migration in biological processes (Fig. 2A), blood microparticle, endoplasmic reticulum lumen, and collagen-containing extracellular matrix in cell components (Fig. 2B), and glycosaminoglycan binding in molecular function (Fig. 2C). Based on the KEGG database, the main pathways involved were complement and coagulation cascades, alcoholic liver disease, phagosome, glycolysis/gluconeogenesis, pentose phosphate pathway, and fructose and mannose metabolism (Fig. 2D).

### Metabolomics analysis of the T2D patients at the baseline and after dapagliflozin treatment

The stability and reliability of metabolomic data were evaluated before data analysis. The overlapping display analysis of QC samples revealed the detection instrument was stable. The violin plot demonstrated that the biases between samples were low (Additional file 2: Fig. S2, Additional file 3: Fig. S3, Additional file 4: Fig. S4, Additional file 5: Fig. S5, Additional file 6: Fig. S6). Collectively, these results indicated the stability and reproducibility of the data. In total, 362 metabolites were identified in method 1 (M1) data, and 415 metabolites were identified in method 2 (M2) data (Additional file 7: Supporting Information). Repeated detected metabolites and a small number of metabolites with coefficients of variation (CV) over 50% in QC were excluded. Finally, a total of 704 metabolites in 23 categories were identified. The levels of the proposed metabolite structural identification were classified by using the report standard by metabolomics Standards Initiative [17]. The resultant metabolomics data are presented in Additional file 11: Table S4. Besides, more detailed chemical information about retention time, exact m/z of adducts, MS2 fragments and so on for the differential metabolites are also presented.

**Fig. 3:**
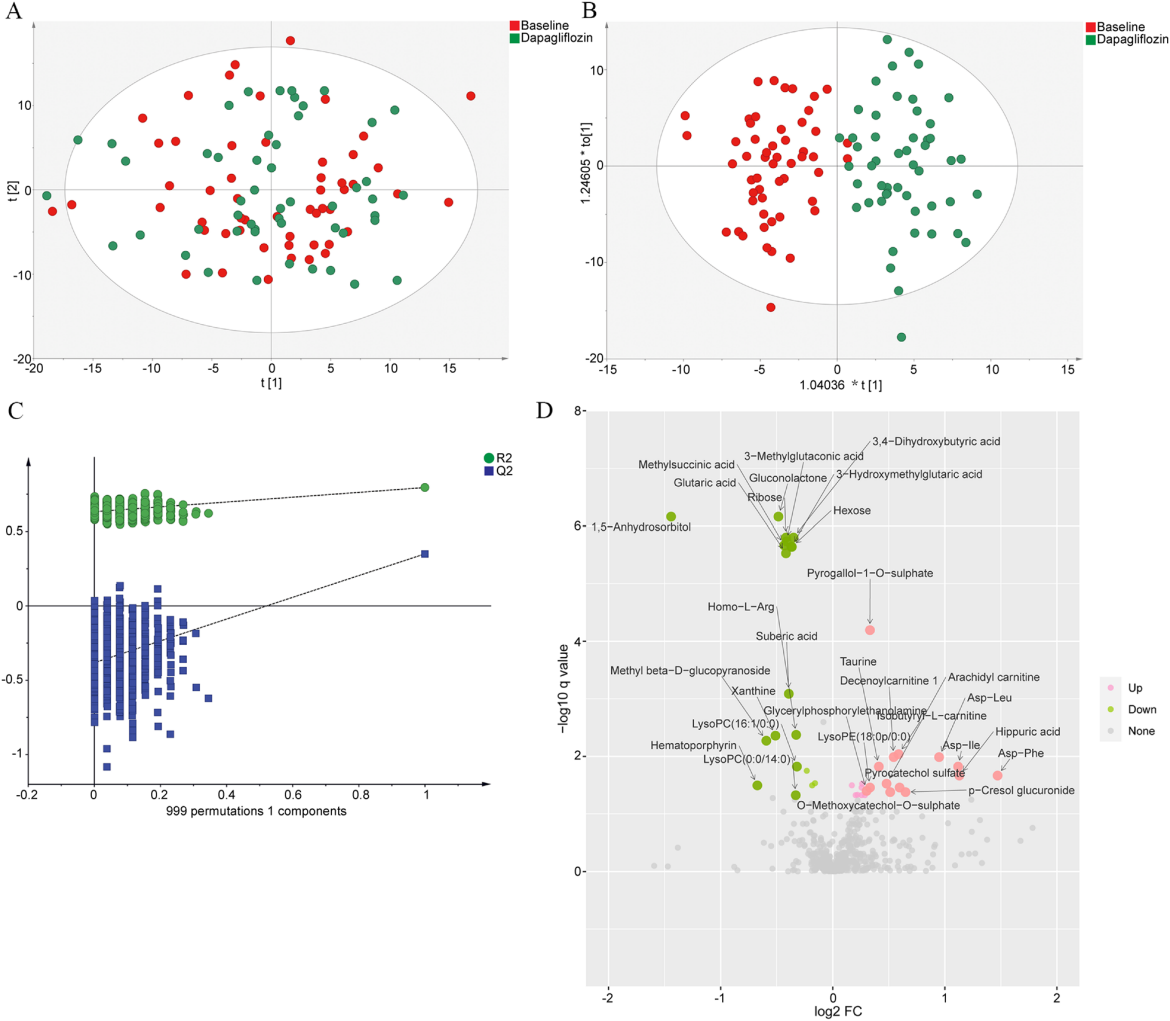
The differential metabolites analysis in the T2D patients at the baseline and after dapagliflozin treatment. **A** PCA score plot of metabolomic data in the T2D patients at the baseline (red dots) and after dapagliflozin treatment (green dots). The cumulative fitness (R2 value) of the PCA model was 0.590. The t [1] and t [2] values in the figures represent the scores of each sample in principal components 1 and 2, respectively. Each dot on the plot represents a sample in the corresponding group. **B** OPLS-DA score plot of metabolomic data in the T2D patients at the baseline (red dots) and after dapagliflozin treatment (green dots) (R2Y = 0.795, Q2 = 0.351). Each dot on the plot represents a sample in the corresponding group. **C** Permutation test of OPLS-DA (R2 intercept = 0.632, Q2 intercept = − 0.382). **D** Volcano plots of the differentially abundant metabolites in the T2D patients after dapagliflozin treatment. The horizontal axis reflects fold change of the metabolites after Log2 logarithmic conversion, and the vertical axis reflects FDR corrected p-value (q-value) after − Log10 logarithmic conversion. In the figure, the pink points indicate significantly differentially increased metabolites, and green points indicate significantly differentially decreased metabolites

Multivariate statistical analyses of these metabolites were performed using PCA and OPLS-DA. First, the clustering of the metabolomic data was assessed using PCA (Fig. 3A). The OPLS-DA analysis indicated clear separations between the baseline (red dots) and after dapagliflozin treatment (green dots) in the T2D patients (R2Y = 0.795, Q2 = 0.351, Fig. 3B). The results of the permutation test strongly indicated that the original model was valid (R2 intercept = 0.632, Q2 intercept = − 0.382, Fig. 3C). A total of 35 metabolites were significantly changed, of which 17 were increased, and 18 were decreased (Additional file 12: Table S5 and Additional file 13: Table S6). All these identified metabolites were further confirmed by the ROC curve analyses (AUC: 0.98, 95% CI 0.96–1.00, Additional file 1: Fig. S1). We also marked differentially abundant metabolites on the volcano plots (Fig. 3D). Dapagliflozin treatment significantly increased several amino acids levels, including aspartyl-leucine (Asp-Leu), aspartyl-isoleucine (Asp-Ile), aspartyl-phenylalanine (Asp-Phe), taurine, and citrulline, and decreased 1,5-anhydrosorbitol, gluconolactone, ribose, hexose, succinic acid, xanthine and uric acid levels (Fig. 3D, Additional file 12: Table S5 and Additional file 13: Table S6).

### Combined analysis of Proteins and Metabolites in the T2D patients after dapagliflozin treatment

**Fig. 4:**
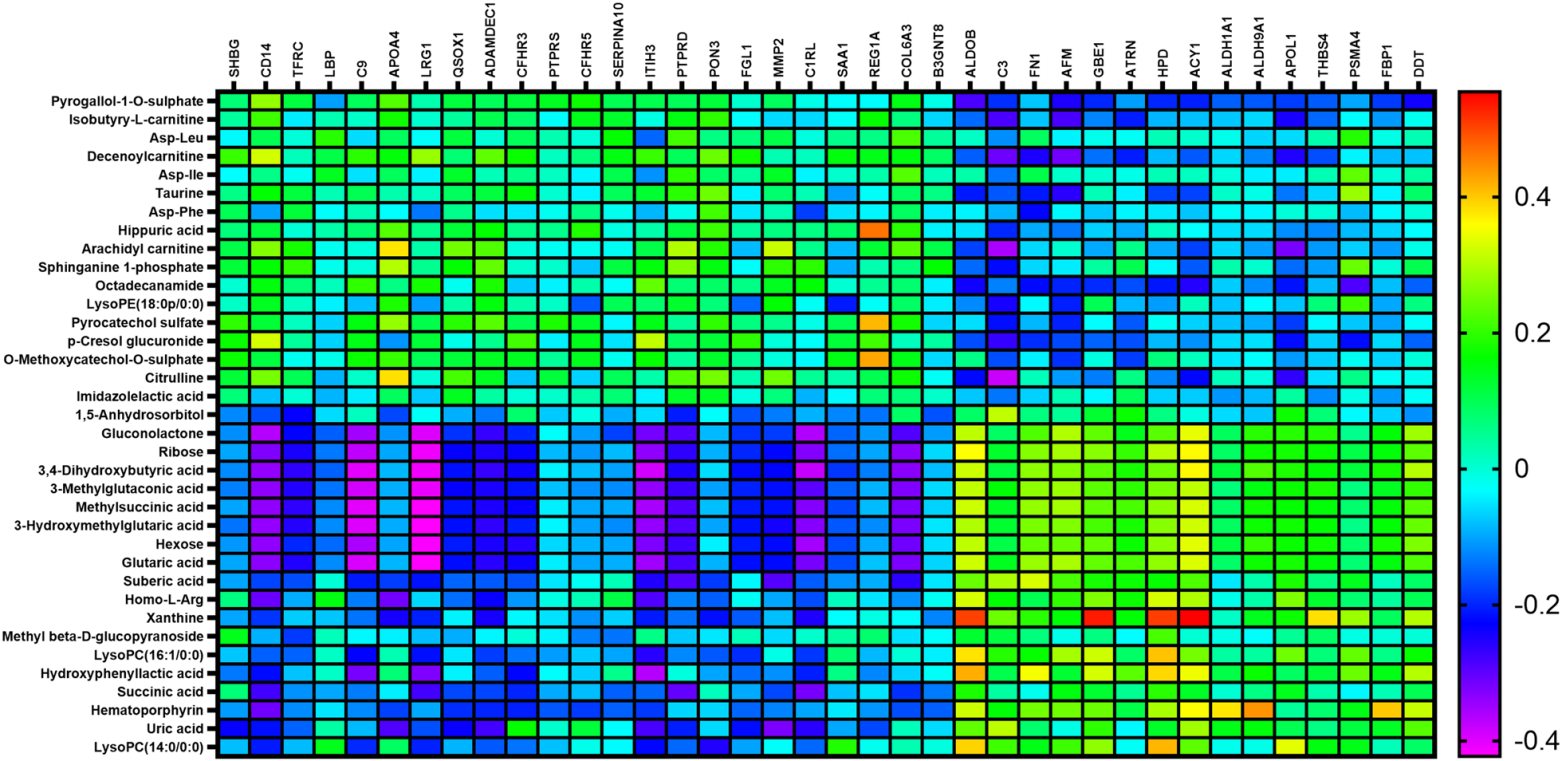
Combined analysis of differential proteins and metabolites in the T2D patients after dapagliflozin treatment. Heat map of correlation between differential protein and differential metabolite expression. The horizontal axis represents proteins, and the vertical axis represents metabolites

We further conducted correlation analysis between the differentially abundant proteins and metabolites to evaluate the protein-metabolites relationship in the T2D patients (Fig. 4 and Additional file 14: Table S7). Xanthine was positively related to ALDOB, 1,4-alpha-glucan-branching enzyme (GBE1), 4-hydroxyphenylpyruvate dioxygenase (HPD), and aminoacylase-1(ACY1). The levels of complement component C9 were negatively associated with various metabolites, such as gluconolactone, ribose, 3,4-dihydroxybutyric acid, 3-methylglutaconic acid, methylsuccinic acid, and 3-hydroxymethylglutaric acid, respectively. The levels of citrulline showed a strong negative correlation with complement component C3. Combined analysis of the differentially abundant proteins and metabolites by KEGG indicated that the main signaling pathways involved were glycolysis/gluconeogenesis and pentose phosphate pathway (Table 2).

**Table 2:** Significant changed pathways of combined analysis of proteins and metabolites by KEGG

NO	Pathway name	Class	Names	*q* value
1	Glycolysis/gluconeogenesis	Protein	Fructose-1,6-bisphosphatase 1(FBP1)	4.71E−02
		Protein	Fructose-bisphosphate aldolase B(ALDOB)	2.36E−06
		Protein	4-trimethylaminobutyraldehyde dehydrogenase (ALDH9A1)	2.24E−02
		Metabolite	Hexose	2.29E−06
2	Pentose phosphate pathway	Protein	Fructose-1,6-bisphosphatase 1(FBP1)	4.71E−02
		Protein	Fructose-bisphosphate aldolase B(ALDOB)	2.36E−06
		Metabolite	Gluconolactone	6.90E−07
		Metabolite	Ribose	1.58E−06
		Metabolite	Hexose	2.29E−06

*q* value is the FDR adjusted *p* value and calculated from the enrichment analysis

## Discussion

The present study showed that dapagliflozin treatment significantly decreased BMI, FBG, HbA1c, and HOMA-IR levels in T2D patients. Multivariate model analysis indicates clear separations of proteomic and metabonomic data between the baseline and after dapagliflozin treatment in T2D patients. A total of 38 differentially abundant proteins including 23 increased and 15 decreased proteins, and 35 differentially abundant metabolites including 17 increased and 18 decreased metabolites, were identified. In addition to influencing glucose metabolism (glycolysis/gluconeogenesis and pentose phosphate pathway), dapagliflozin significantly increased SHBG, TFRC, ADAMDEC1, and APOA4 levels, and decreased complement C3, FN1, AFM, ATRN, xanthine, and uric acid levels.

Our study was consistent with the previous studies and showed that SGLT2i significantly increased TFRC levels in T2D patients [18, 19]. TFRC can transfer Fe^2+^ into cytoplasm, and increased cytosolic Fe^2+^ further facilitates the synthesis of ATP in cardiomyocytes and heme in erythroid precursors, which has been believed as a possible mechanism for the beneficial effect on major heart failure events of SGLT2i [20, 21]. Interestingly, the present study found that SHBG was significantly increased in the T2D patients after dapagliflozin treatment. SHBG is produced in the liver and plays an important role in regulating the bioavailability of testosterone and estradiol [22]. Insulin resistance and hyperinsulinemia cause decreased synthesis and production of SHBG by inhibiting hepatocyte nuclear factor 4α (HNF-4α) expression in the liver [23, 24]. Dapagliflozin treatment diminished FINS and increased insulin sensitivity, which might explain the increase of SHBG after dapagliflozin treatment. It is worth noting that dapagliflozin treatment significantly increased ADAMDEC1 and APOA4 levels of the T2D patients in the present study. ADAMDEC1 was the most significant differential protein in the T2D patients after dapagliflozin treatment in the present study. Both ADAMDEC1 and APOA4 act as anti-inflammatory proteins and are mainly synthesized in the gastrointestinal tract in humans [25–27]. *Adamdec1*-deficient mice manifest elevated inflammatory response, exaggerated bacterial translocation, and higher mortality during chemical or bacterial-induced colitis [25]. A recent study showed that APOA4 had an anti-inflammatory effect in alleviating dextran sulfate sodium-induced colitis in mice [28]. APOA4 is also involved in regulating lipid absorption and metabolism, and glucose homeostasis [29, 30]. Decreased circulating APOA4 concentration is associated with a higher risk of coronary artery disease and diabetes [29, 31]. Therefore, these results suggested that dapagliflozin might regulate the function and immune status of the gastrointestinal tract. Dapagliflozin treatment significantly decreased FBP1 and ALDOB levels in the T2D patients in the present study. Both FBP1 and ALDOB were primarily abundant in the liver and kidney, and play key roles in gluconeogenesis and glycolysis [32, 33]. Previous studies have shown that plasma ALDOB levels were elevated in obesity and non-alcoholic fatty liver disease (NAFLD) in both humans and mice [34, 35]. Metformin decreased hepatic glucose production by inhibiting FBP [36]. Notably, the present study also found dapagliflozin treatment significantly reduced the complement C3 levels of the T2D patients. Complement C3 leads to the development and deterioration of metabolic disorders and cardiovascular disease by promoting insulin resistance and inflammation [37, 38]. Many studies have shown that complement C3 levels were higher in multiple metabolic diseases, including obesity, diabetes, and NAFLD, and an energy-restricted diet decreased plasma C3 levels in men with obesity [39–42]. A recent study found that dapagliflozin treatment decreased mRNA and protein expression of C3 in the kidney of the db/db mice [43]. These changes in proteins might be associated with the beneficial effect of dapagliflozin on cardiovascular and renal outcomes.

Enrichment of pathways in the KEGG database further supports the beneficial effects of dapagliflozin. Pathway analysis using KEGG annotation showed that three of them were related to glucose metabolism. The decrease of metabolites in the glycolysis/gluconeogenesis pathway may be due to SGLT2i directly inhibiting the reabsorption of glucose, fatty acids and other fuel sources in renal tubules [44]. SGLT2i inhibits the activation of glycolysis and contributes to the reduction of subsequent metabolic pathways related intermediates, including the pentose phosphate pathway, the hexosamine pathway, and lipid synthesis [45]. Endogenous fructose can be increased in response to hyperglycemia induction. SGLT2i also reduces the amount of glucose converted to fructose while blocking glucose absorption into the proximal tubule [45].

In the present study, we found that the function of dapagliflozin was enriched in leukocyte chemotaxis and myeloid leukocyte migration, which has been reported in other diseases, such as glycogen storage disease type Ib (GSD-Ib) [46]. The reason for this change might be that SGLT2i could reduce neutrophil 1,5-anhydroglucitol-6-phosphate (1,5AG6P), a powerful inhibitor of hexokinases, which further affects the glycolysis and energy metabolism of bone marrow cells [46]. The findings suggested a potential anti-inflammatory effect of dapagliflozin.

In addition, there are several previous evidences showing that dapagliflozin plays a protective role in the kidney and heart through lysosomal, phagocytic and autophagy pathways [47–49]. The protective effect of empagliflozin in non-diabetic chronic kidney disease (CKD) mice is partially mediated by the complement system, as demonstrated by RNA sequencing [50]. Our study found that dapagliflozin also affected complement components and the coagulation cascade, which were related to the reduction of atherosclerotic plaque [51]. Additionally, enrichment analysis of cell components found that the potential targets were mainly distributed in blood microparticle and extracellular matrix, which are related to the function of dapagliflozin in protecting vascular endothelium [52]. Animal studies have demonstrated that the functional benefit of dapagliflozin on the kidney was accompanied by a reduction in extracellular matrix accumulation [53]. The extracellular matrix, composed of proteins (especially collagen) and glycosaminoglycans (mainly proteoglycans), not only provides the necessary physical scaffold for cellular components, but also provides an environment for cell morphogenesis, differentiation, and homeostasis.

Therefore, these results suggested that in addition to the hypoglycemic effect, dapagliflozin can also provide multi-organ protection, and reveal the potential underlying mechanisms.

The present study showed that 1,5-anhydrosorbitol was the most differentially abundant metabolite in T2D patients after dapagliflozin treatment. 1,5-anhydrosorbitol is a naturally dietary inert polyol [54, 55]. In the kidney, 1,5-anhydrosorbitol competes with glucose for reabsorption [54, 55]. Therefore, the decreased circulating 1,5-anhydrosorbitol levels were associated with increased urinary glucose excretion. We also found dapagliflozin treatment significantly decreased other monosaccharides levels including ribose (five-carbon sugar) and hexose (six-carbon sugar) in T2D patients. In addition, a decrease in succinic acid was also observed after dapagliflozin treatment. Succinic acid is a dicarboxylic acid and is produced in the tricarboxylic acid cycle in mitochondria [56]. These results suggested that dapagliflozin significantly influenced glucose metabolism. As we all know, dapagliflozin treatment generates a state of negative energy balance by increasing urinary glucose excretion [2–4]. In the present study, several amino acids, including Asp-Leu, Asp-Ile, and Asp-Phe, were significantly increased in T2D patients after dapagliflozin treatment, which might be associated with increased protein catabolism in negative energy balance. Interestingly, we also found increased taurine levels in the T2D patients after dapagliflozin treatment. Low circulating taurine has been observed in many diseases, such as diabetes and heart failure [57, 58]. In animal models, taurine could improve adipocyte hypertrophy, promote thermogenesis, and inhibit high-fat diet induced weight gain [59]. In addition, taurine has been proven to increase the activity of respiratory chain complexes I, accelerate ATP generation, and further improve cardiac contractility [60–62]. The present study was consistent with many previous studies and showed that SGLT2i significantly decreased uric acid in T2D patients [63, 64]. SGLT2i may directly or indirectly reduce circulating uric acid via several mechanisms including facilitating urinary uric acid excretion, inhibiting xanthine oxidase via stimulating sirtuin-1, and suppressing inflammatory response [65]. Uric acid has long been considered an independent predictor of worse outcomes in patients with heart failure [66]. The decrease in uric acid was also believed to partly mediate the beneficial effect of SGLT2i on cardiovascular and kidney endpoint events [63, 64, 67]. Therefore, increased taurine and reduced uric acid might be associated with the beneficial effect of dapagliflozin on cardiovascular and renal outcomes.

The integration of metabolomics and proteomics provides a comprehensive understanding of metabolic changes after dapagliflozin treatment, which is difficult to achieve with one platform alone. Dapagliflozin reduces circulating uric acid levels by inhibiting xanthine oxidase. Many patients with hyperuricemia are accompanied by obesity, diabetes, hypertension, and chronic kidney diseases. When the level of circulating uric acid is decreased, ALDOB related to fructose metabolism [68], GBE1 related to glucose hydrolysis [69], and ACY1 related to amino acid metabolism and insulin homeostasis are decreased [70], which indicates that dapagliflozin treatment not only reduces uric acid but also improves the occurrence of complications. HPD mediates tyrosine metabolism, and disturbed tyrosine catabolism is associated with the deterioration of liver function [71]. The positive correlation between xanthine and HPD indicates that the stability of uric acid levels changes synchronously with the improvement of liver function. SGLT2i is effective in alleviating chronic kidney diseases, partly through downregulation of the complement system. The multi-organ benefits of dapagliflozin can significantly promote energy metabolism. The differential metabolites identified by our study are located in the tricarboxylic acid cycle (glucolactone, 3-methylpentenoic acid, methylsuccinic acid) [72–74], ribose and urea cycle (citrulline) [75], and these metabolites are negatively correlated with the complement system, which are associated with the beneficial effects of dapagliflozin on the kidney and the improvement of metabolism. SGLT2i reduce glucose load by increasing glycosuria, thereby causing changes in glucose metabolism. SGLT2i inhibit hepatic glycolysis by inhibiting pyruvate kinase [76]. Animal experiments have also demonstrated that the down-regulation of Phosphoenolpyruvate carboxykinase (PEPCK) and Glucose-6-phosphatase (G6Pase) activities by Adenosine 5 ‘-monophosphate (AMP)-activated protein kinase/ cAMP-response element binding protein (AMPK/CREB) signaling pathway can inhibit hepatic gluconeogenesis, and the activation of AMPK/ Glycogen synthase kinase 3β (GSK3β) signaling pathway in vivo can promote glycogen synthesis [77]. This is consistent with our KEGG enriched pathways. These associations provide valuable insights into the complex relationships between differential proteins and metabolites. Based on the results of this integrated analysis, we strongly recommend prioritizing future studies of the relationship among these metabolites/proteins. These studies have the potential to shed light on key mechanisms underlying the multiorgan metabolic benefits of dapagliflozin.

The present study has some limitations. First, this study was a single-center study with a relatively small sample size. Hence, some confounders might influence the results. Second, this study was lack of a healthy control group with matched age, gender, and BMI. Thus, it was unclear whether metabolite or protein differed between healthy control and T2D patients. Third, according to the Human metabolomics Database, there are many isomers of hexose with Chemical formula C6H12O6, of which D-glucose is the predominant one with the highest concentration, and myo-inositol is another dominating hexose in human blood. Due to the technical limitation of the analytical method employed in current research, the isomers of hexose cannot be distinguished by using reverse phase separation, in consequence, this metabolic feature was chemically structurally annotated as hexose (and primarily, a sum of glucose and myo-inositol). Finally, it will be more convincing if we add the ROC analysis using a new batch of samples. However, our sample size was too small for a validation data set, so ROC curve analysis could not be performed. Our current study is only a preliminary report that serves as a basis for future research. Nevertheless, an integrated evaluation of proteomics and metabolomics provides a valuable direction for explaining the beneficial effect of SGLT2i. Further randomized-controlled studies with a large sample size are needed to confirm our results.

## Conclusions

The present study showed that serum proteomics and metabolomics were significantly changed after dapagliflozin treatment. As a hypoglycemic agent, dapagliflozin treatment mainly influenced glycolysis/gluconeogenesis and pentose phosphate pathway. Moreover, dapagliflozin increased SHBG, TFRC, and anti-inflammatory proteins (ADAMDEC1 and APOA4) levels, and decreased C3, xanthine, and uric acid levels. These changes in proteins and metabolites might be associated with the beneficial effect of dapagliflozin on cardiovascular and renal outcomes.

### Supplementary Information

**Additional file 1: Figure S1.** The ROC curve analyses for all identified proteins and metabolites.

**Additional file 2: Figure S2.** PCA score plot of Quality control (QC) samples. The t [1] and t [2] values in the figures represent the scores of each sample in principal components 1 and 2, respectively. Black dots: QC samples; Green dots: samples tested in this project.

**Additional file 3: Figure S3.** Time series plot of principal component 1 during PCA analytical batch.

**Additional file 4: Figure S4.** Metabolite intensity RSD% distribution in QCs samples. The horizontal axis represents RSD% distribution, and the vertical axis represents the metabolites percentage in the RSD% distribution. The RSD% of all metabolites is < 30%. RSD: relative standard deviation.

**Additional file 5: Figure S5.** Spearman correlation analysis of the first and last QC samples in the analysis batch. High correlation indicated high data quality of acquired untargeted metabolomic data.

**Additional file 6: Figure S6.** Violin plot of CV% for all quantified metabolites in each group. CV: coefficients of variation.

**Additional file 7.** Supporting Information. The main parameters for Proteomics and Metabolomics.

**Additional file 8: Table S1.** Increased proteins in the T2D patients after dapagliflozin treatment.

**Additional file 9: Table S2.** Decreased proteins in the T2D patients after dapagliflozin treatment.

**Additional file 10: Table S3.** The Gene Ontology (GO) and the Kyoto Encyclopedia of Genes and Genomes (KEGG) pathway of the 38 differentially expressed proteins.

**Additional file 11: Table S4.** Metabolomics result of the T2D patients at the baseline and after dapagliflozin treatment.

**Additional file 12: Table S5.** Increased metabolites in the T2D patients after dapagliflozin treatment.

**Additional file 13: Table S6.** Decreased metabolites in the T2D patients after dapagliflozin treatment.

**Additional file 14: Table S7.** The correlation analysis between the differentially abundant proteins and metabolites.

## Data Availability

The datasets presented in this study can be found in online repositories. The name of the repository and accession numbers can be found below: iProX; IPX0007492000 and MetaboLights; MTBLS8885.

## Abbreviations

SGLT2: Sodium-glucose co-transporter 2
T2D: Type 2 diabetes
HbA1c: Hemoglobin A1c
BMI: Body Mass Index
TG: Serum triglycerides
TC: Total cholesterol
HDL-C: High-density lipoprotein cholesterol
LDL-C: Low-density lipoprotein cholesterol
NEFA: Nonesterified fatty acid
FBG: Fasting plasma glucose
FINS: Fasting plasma insulin
CREA: Creatinine
HOMA-IR: Homeostasis model assessment of insulin resistance
eGFR: Estimated glomerular filtration rate
MS: Mass spectrometry
ACN: Acetonitrile
FA: Formic acid
SDS: Sodium dodecyl sulfate
NH4HCO3: Ammonium biocarbonate
TFA: Trifluoroacetic acid
DTT: DT-Dithiothreitol
IAA: Iodoacetamide
LysC: Lysyl endopeptidase
LC–MS: Liquid chromatography-mass spectrometry
DIA: Data-independent acquisition
QC: Quality control
FDR: False discovery rate
PCA: Principal component analysis
OPLS-DA: Orthogonal partial least square discriminant analysis
FC: Fold change
GO: Gene Ontology
KEGG: Kyoto Encyclopedia of Genes and Genomes
SHBG: Sex hormone-binding globulin
TFRC: Transferrin receptor protein 1
ADAMDEC1: Disintegrin and metalloprotease-like decysin-1
APOA4: Apolipoprotein A-IV
FBP1: Fructose-1,6-bisphosphatase 1
ALDOB: Fructose-bisphosphate aldolase B
FN1: Fibronectin
AFM: Afamin
ATRN: Attractin
CV: Coefficients of variation
Asp-Leu: Aspartyl-leucine
Asp-Ile: Aspartyl-isoleucine
Asp-Phe: Aspartylphenylalanine
GBE1: 1,4-Alpha-glucan-branching enzyme
HPD: Hydroxyphenylpyruvate dioxygenase
ACY1: Aminoacylase-1
HNF-4α: Hepatocyte nuclear factor 4α
