# Supplementary figures and images for: Effect of dapagliflozin on proteomics and metabolomics of serum from patients with type 2 diabetes
Source: Diabetol Metab Syndr. 2023 Dec 4;15:251. doi: 10.1186/s13098-023-01229-0 (PMC10694884; doi:10.1186/s13098-023-01229-0)

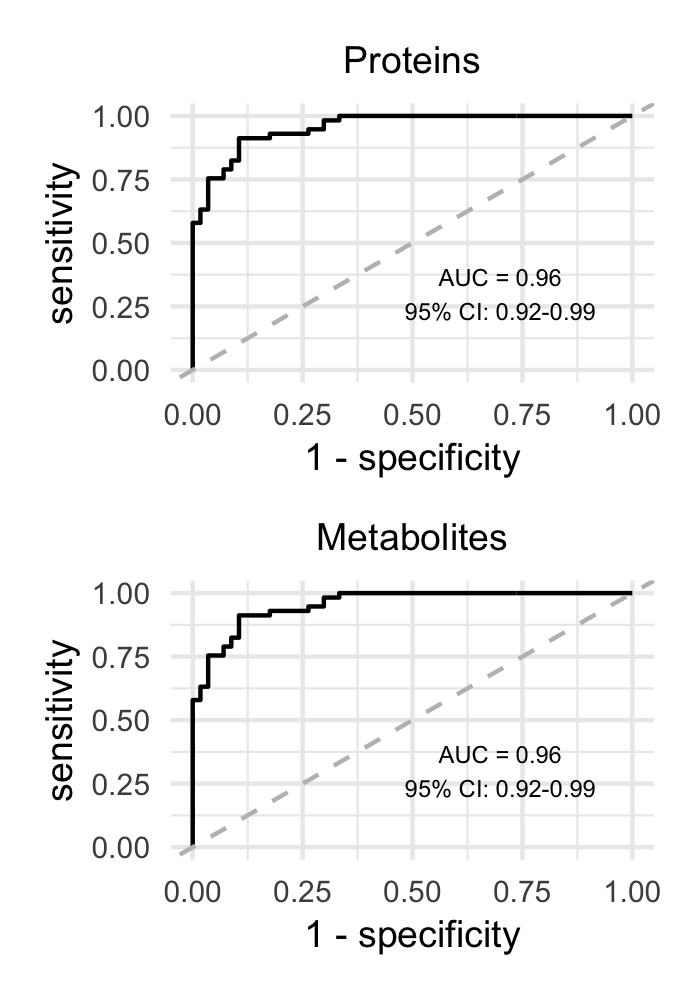

Supplement: Supplementary file 1 — Additional file 1: Figure S1. The ROC curve analyses for all identified proteins and metabolites. [file 13098_2023_1229_MOESM1_ESM.tiff]

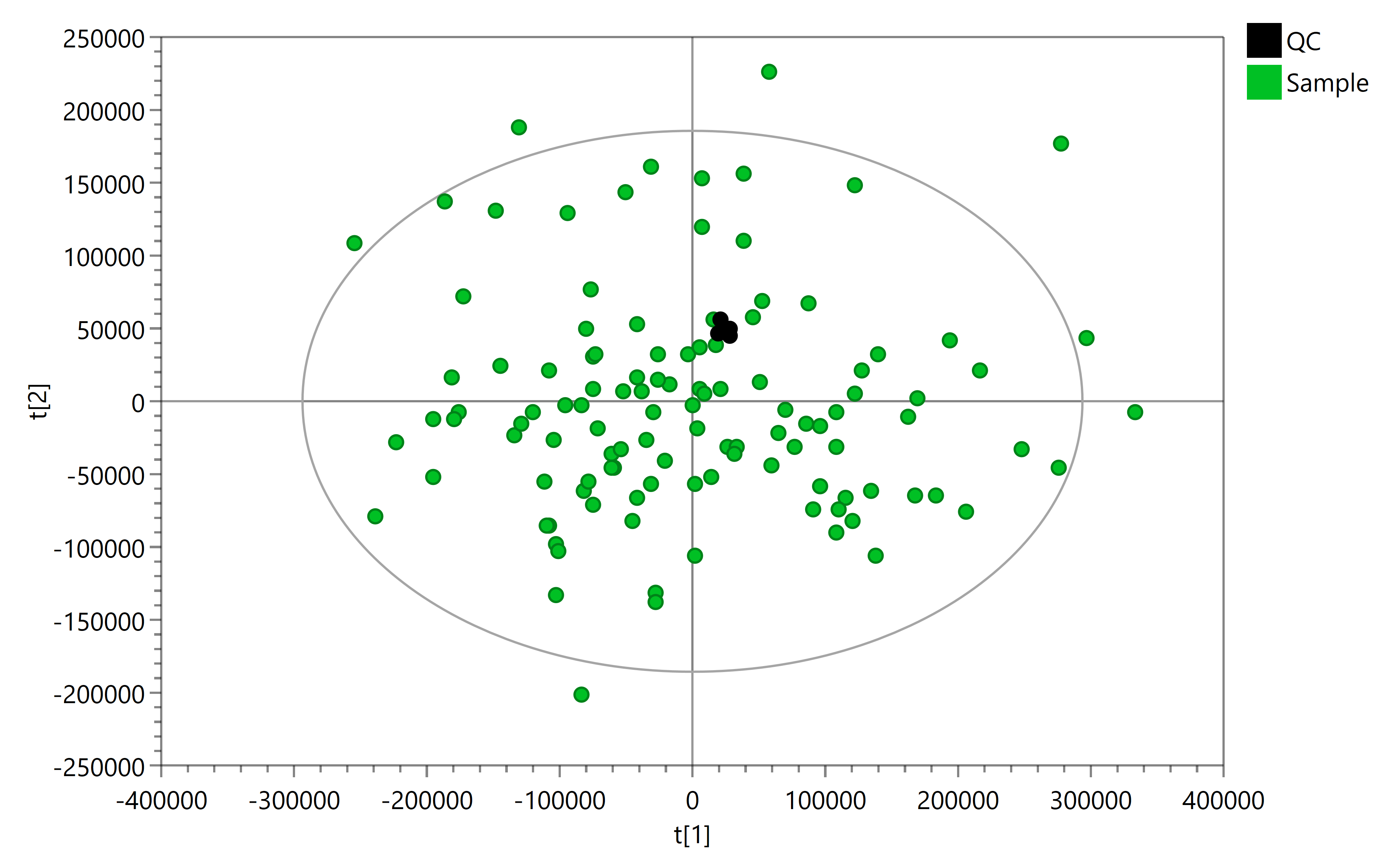

Supplement: Supplementary file 2 — Additional file 2: Figure S2. PCA score plot of Quality control (QC) samples. The t [1] and t [2] values in the figures represent the scores of each sample in principal components 1 and 2, respectively. Black dots: QC samples; Green dots: samples tested in this project. [file 13098_2023_1229_MOESM2_ESM.tif]

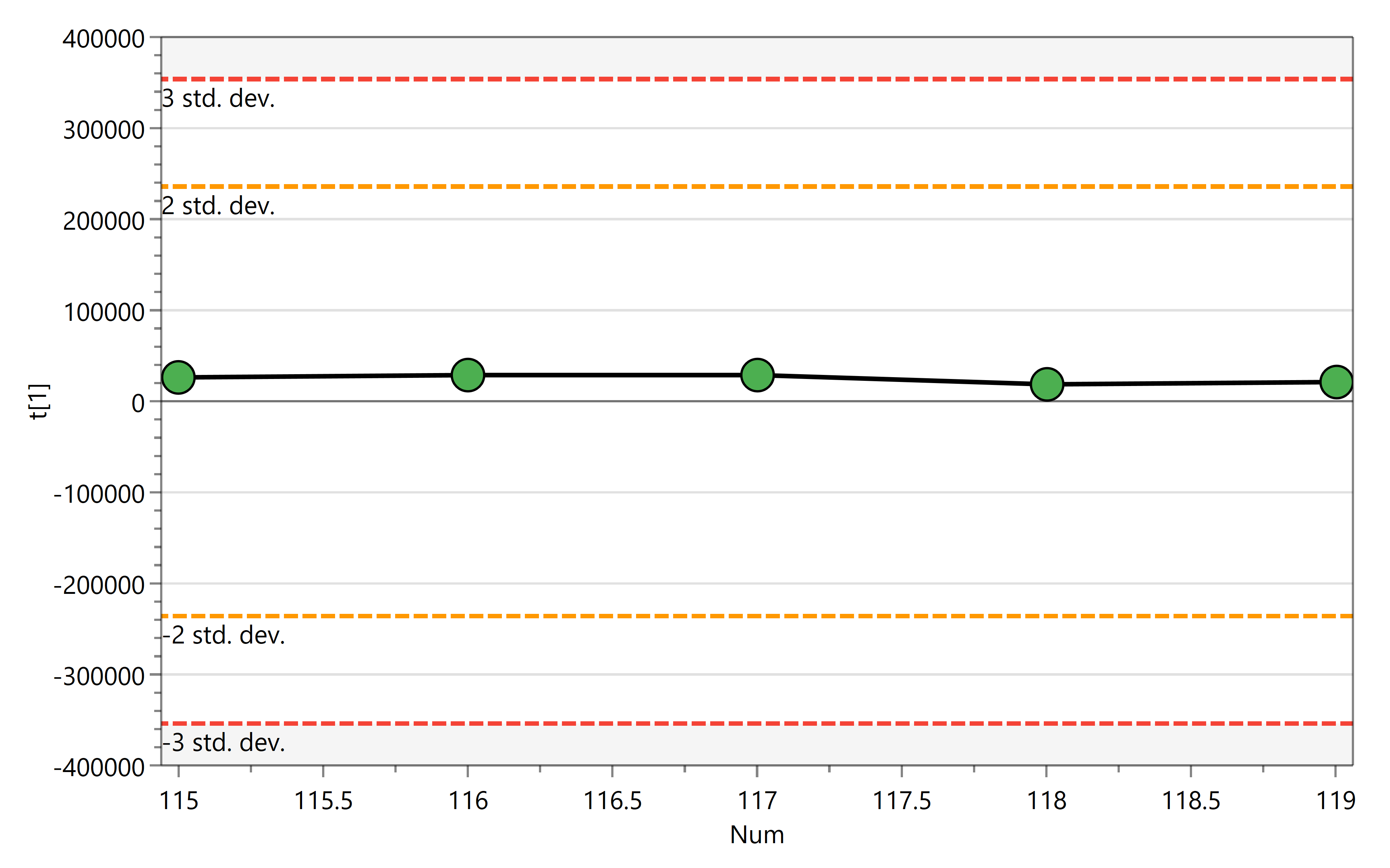

Supplement: Supplementary file 3 — Additional file 3: Figure S3. Time series plot of principal component 1 during PCA analytical batch. [file 13098_2023_1229_MOESM3_ESM.tif]

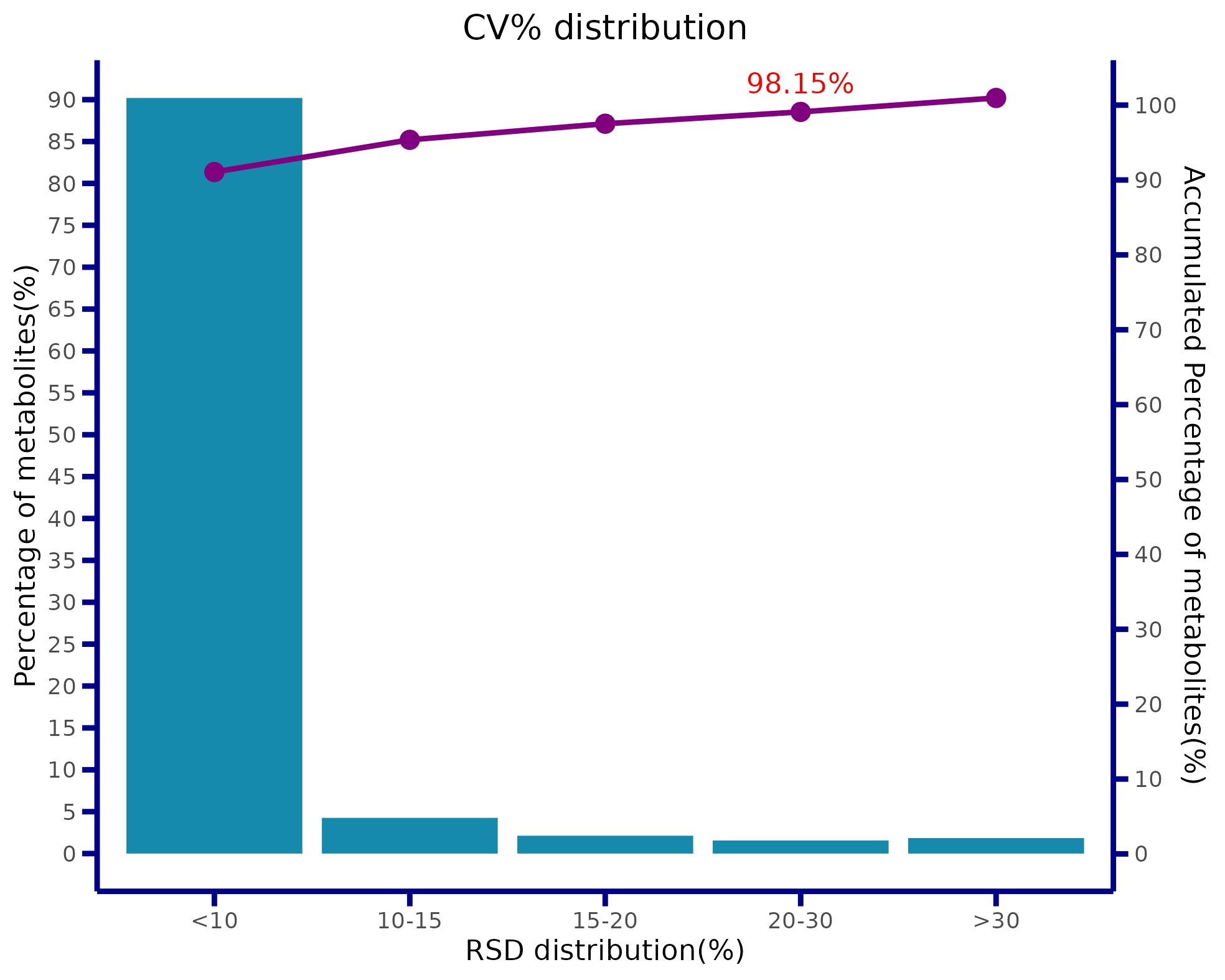

Supplement: Supplementary file 4 — Additional file 4: Figure S4. Metabolite intensity RSD% distribution in QCs samples. The horizontal axis represents RSD% distribution, and the vertical axis represents the metabolites percentage in the RSD% distribution. The RSD% of all metabolites is < 30%. RSD: relative standard deviation. [file 13098_2023_1229_MOESM4_ESM.tiff]

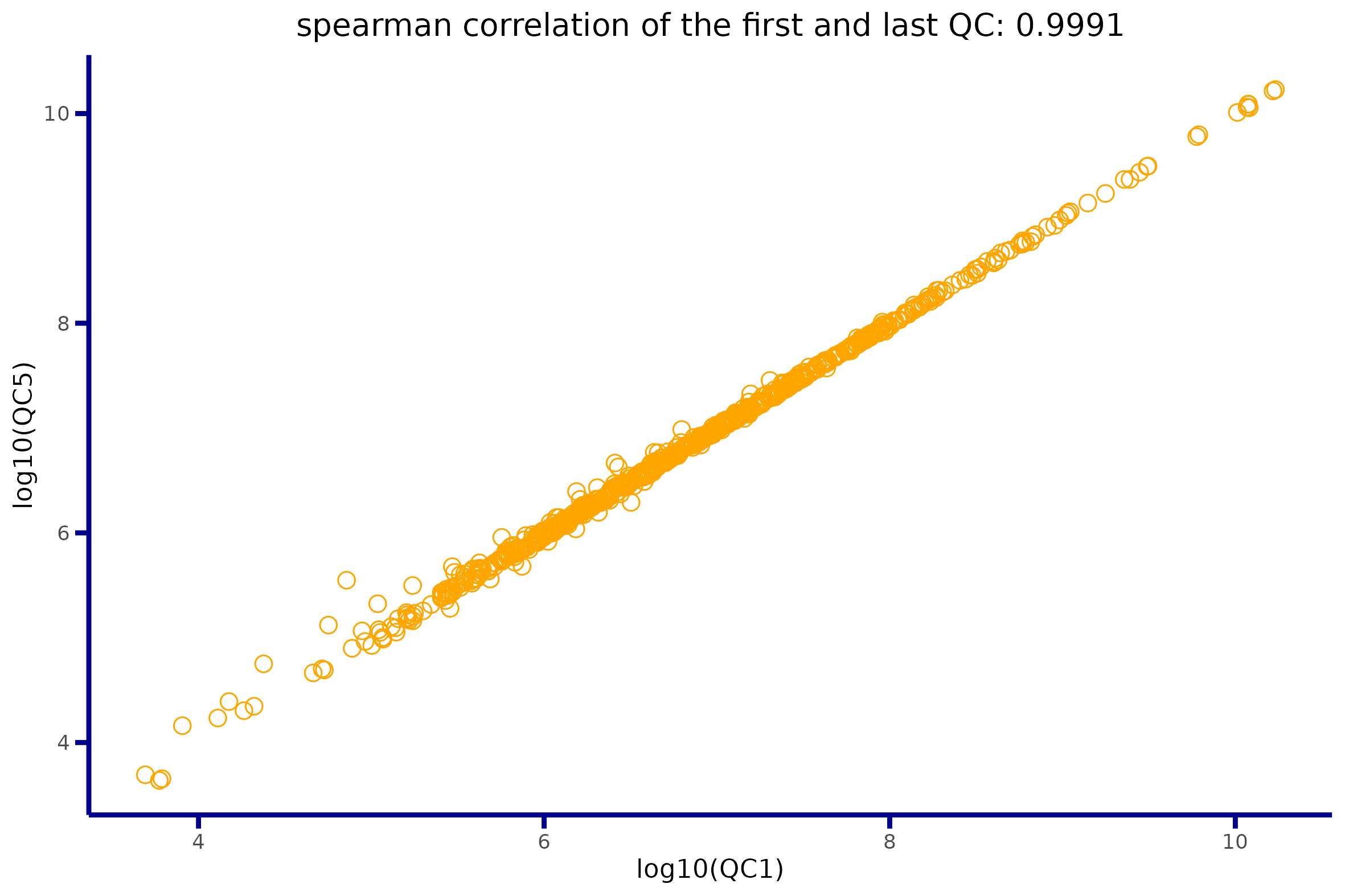

Supplement: Supplementary file 5 — Additional file 5: Figure S5. Spearman correlation analysis of the first and last QC samples in the analysis batch. High correlation indicated high data quality of acquired untargeted metabolomic data. [file 13098_2023_1229_MOESM5_ESM.tiff]

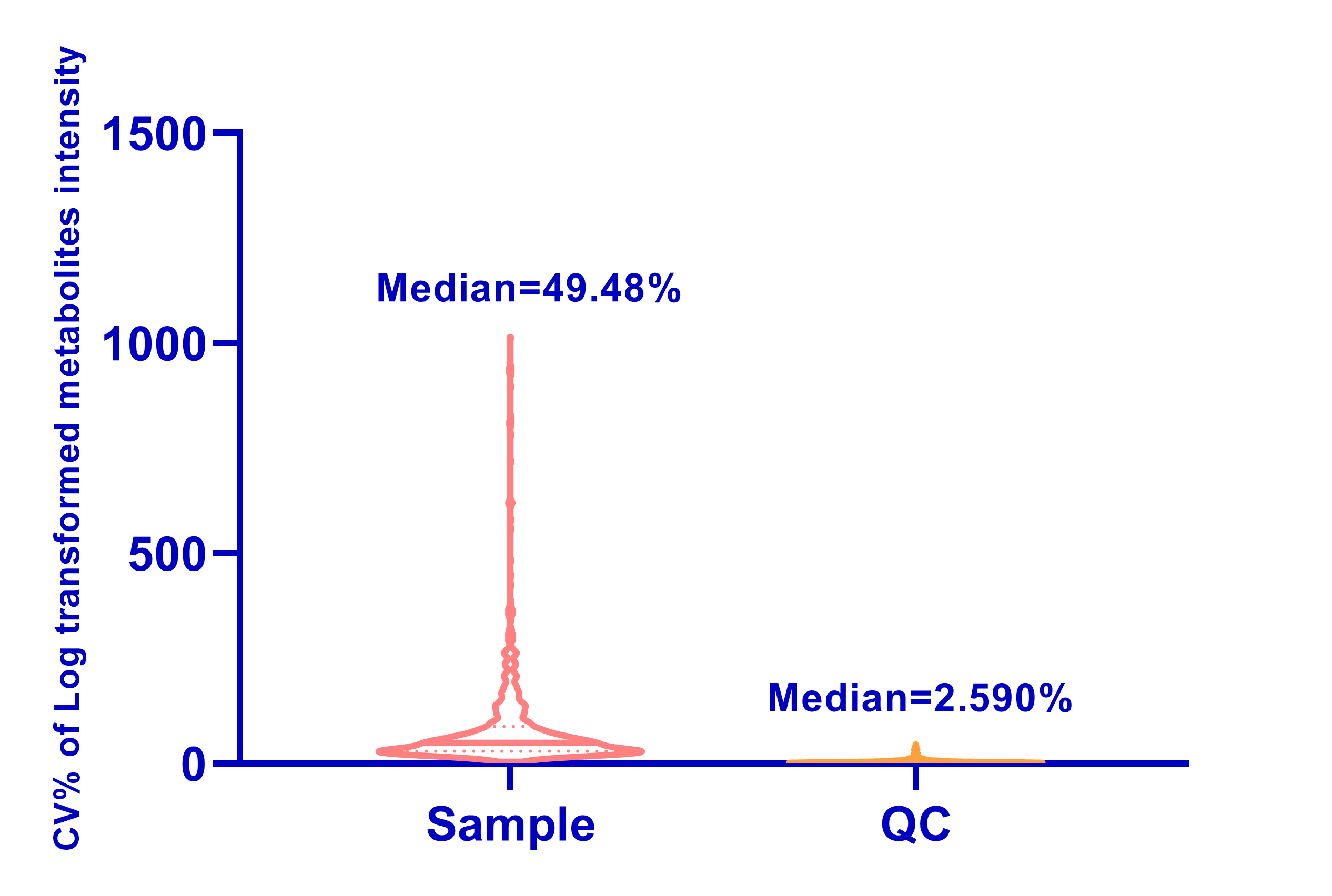

Supplement: Supplementary file 6 — Additional file 6: Figure S6. Violin plot of CV% for all quantified metabolites in each group. CV: coefficients of variation. [file 13098_2023_1229_MOESM6_ESM.tif]
