# Supplementary material for: Effect of dapagliflozin on proteomics and metabolomics of serum from patients with type 2 diabetes
Source: Diabetol Metab Syndr. 2023 Dec 4;15:251. doi: 10.1186/s13098-023-01229-0 (PMC10694884; doi:10.1186/s13098-023-01229-0)
Supplement: Supplementary file 7 — Additional file 7. Supporting Information. The main parameters for Proteomics and Metabolomics. [file 13098_2023_1229_MOESM7_ESM.docx]

**Additional file 7**

1. The main parameters of LC-MS analysis for Proteomics

nanoLC-UltiMate 3000 RSLCnano System, two Column Mode: Trap Column (Acclaim PepMap C18, 3μm, 100Å, 75μm x 2 cm), Analytical Column (Acclaim PepMap C18, 2μm, 100Å, 75μm x 25 cm); mobile phase: A: 0.1% Formic acid in water; B: 0.1% Formic acid in 80% Acetonitrile; Gradient: 3% - 6% B in 3 min, 8% - 30% B in 95 min, 30% - 99% B in 4 min, 99% - 99% B in 5 min; flow rate: 300 nL/min.

GPF-DIA for chromatography library: Mass spectrometer: Thermo Scientific Orbitrap Fusion Lumos; spray voltage: 2.1 kV; capillary temperature: 300 °C; S-lens: 50%; collision energy: 32% HCD; resolution setting: full ms 60,000@m/z 200, DIA scans 30,000@m/z 200; Max IT: full MS 20ms, full MS/MS 54ms; parent ion scanning range: m/z 400-550, 550-700, 700-850, 850-1000 for each injection; product ion scanning range: start from m/z 200; number of windows: 80; Isolation window: 3 Da.

DIA of samples for quantification: Q Exactive HFX; spray voltage: 2.1 kV; capillary temperature: 300 °C; S-lens: 50%; collision energy: 32% HCD; resolution setting: full ms 60,000@m/z 200, DIA scans 30,000@m/z 200; Max IT: full MS 20ms, full MS/MS 54ms; parent ion scanning range: m/z 350-1200; product ion scanning range: start from m/z 200; number of windows: 80; Isolation window 10 Da.

2. The main parameters of Ultra-high performance liquid chromatography-high resolution mass spectrometry analysis for Metabolomics

The metabolite extracts were analyzed with two complementary analytical methods (method 1 and 2) basing on reversed-phase chromatography separation mode. Acidic binary mobile phases and positive electrospray ionization mode were employed for method 1, metabolites were separated by using an ACE C18-PFP column (Advanced Chromatography Technologies Ltd, Aberdeen, Scotland, 2.0 μm, 2.1 × 100 mm), and eluted by 0.1% FA in water and ACN using linear gradient ramping from 2% to 98% organic mobile phase for 10 min. Meanwhile, other metabolite extracts were eluted by water and ammonium ACN/methanol containing ammonium bicarbonate buffer and separated on an Acquity^TM^ HSS C_18_ column (Waters Corporation, Milford, USA, 1.8 μm, 2.1 × 100 mm) and detected by negative electrospray ionization for method 2. The gradient was used as follow: 0 min 2% organic phase ramped to 100% in 10 min, and other 5 min was used for column washing and equilibrating. The flowrate, injection volume and column temperature were set at 0.4 mL/min, 5 μL and 50℃ for both two methods, respectively.

For method 1 and 2, the quadrupole-Orbitrap mass spectrometer was conducted under identical ionization parameters with a heated electrospray ionization source except ionization voltage(4.0 kV for positive mode and 3.5 KV for negative mode) including sheath gas 45 arb, auxiliary gas 10 arb, heater temperature 355℃, capillary temperature 320℃ and S-Lens RF level 55%.

The metabolite extracts were employed with full scan mode, 70,000 full widths at half-maximum (FWHM) resolution with automatic gain control (AGC) with 3e6 and max injection time with 200 ms. FWHM 70~1000 m/z scan range was acquired. QC samples were repeatedly injected to acquired Top 10 data dependent MS^2^ spectra (full scan-ddMS^2^) for comprehensive metabolite structural annotation. 17,500 FWHM resolution setting were used for full MS/MS data acquisition. Apex trigger, dynamic exclusion and isotope exclusion was conducted, and precursor isolation window was set at 1.0 Da. Stepped normalized collision energy was employed for collision induced disassociation of metabolite using ultra-pure nitrogen as fragmentation gas.

3. Additional files Figures

Additional file 1: Figure S1. The ROC curve analyses for all identified proteins and metabolites.

Additional file 2: Figure S2. PCA score plot of Quality control (QC) samples. The t [1] and t [2] values in the figures represent the scores of each sample in principal components 1 and 2, respectively. Black dots: QC samples; Green dots: samples tested in this project.

Additional file 3: Figure S3. Time series plot of principal component 1 during PCA analytical batch.

Additional file 4: Figure S4. Metabolite intensity RSD% distribution in QCs samples. The horizontal axis represents RSD% distribution, and the vertical axis represents the metabolites percentage in the RSD% distribution. The RSD% of all metabolites is < 30%. RSD: relative standard deviation.

Additional file 5: Figure S5. Spearman correlation analysis of the first and last QC samples in the analysis batch. High correlation indicated high data quality of acquired untargeted metabolomic data.

Additional file 6: Figure S6. Violin plot of CV% for all quantified metabolites in each group. CV: coefficients of variation.

4. Additional files Tables

Additional file 8: Table S1. Increased proteins in the T2D patients after dapagliflozin treatment.

Additional file 9: Table S2. Decreased proteins in the T2D patients after dapagliflozin treatment.

Additional file 10: Table S3. The Gene Ontology (GO) and the Kyoto Encyclopedia of Genes and Genomes (KEGG) pathway of the 38 differentially expressed proteins.

Additional file 11: Table S4. Metabolomics result of the T2D patients at the baseline and after dapagliflozin treatment.

Additional file 12: Table S5. Increased metabolites in the T2D patients after dapagliflozin treatment.

Additional file 13: Table S6. Decreased metabolites in the T2D patients after dapagliflozin treatment.

Additional file 14: Table S7. The correlation analysis between the differentially abundant proteins and metabolites.
