# Supplementary material for: Effect of dapagliflozin on proteomics and metabolomics of serum from patients with type 2 diabetes
Source: Diabetol Metab Syndr. 2023 Dec 4;15:251. doi: 10.1186/s13098-023-01229-0 (PMC10694884; doi:10.1186/s13098-023-01229-0)
Supplement: Supplementary file 8 — Additional file 8: Table S1. Increased proteins in the T2D patients after dapagliflozin treatment. [file 13098_2023_1229_MOESM8_ESM.docx]

Additional file 8: Table S1. Increased proteins in the T2D patients after dapagliflozin treatment

| **Protein** | **log2FC** | ***FC*** | ***p value*** | ***q value*** |
| --- | --- | --- | --- | --- |
| SHBG | 0.51 | 1.42 | 8.15E-09 | 3.34E-06 |
| CD14 | 0.28 | 1.22 | 9.31E-06 | 1.53E-03 |
| TFRC | 0.35 | 1.28 | 1.83E-05 | 2.50E-03 |
| LBP | 0.42 | 1.34 | 2.47E-05 | 2.89E-03 |
| C9 | 0.28 | 1.22 | 1.34E-04 | 9.14E-03 |
| APOA4 | 0.30 | 1.24 | 1.63E-04 | 1.03E-02 |
| LRG1 | 0.22 | 1.17 | 3.05E-04 | 1.47E-02 |
| QSOX1 | 0.52 | 1.43 | 3.78E-04 | 1.72E-02 |
| ADAMDEC1 | 1.73 | 3.31 | 4.40E-04 | 1.80E-02 |
| CFHR3 | 0.24 | 1.18 | 4.23E-04 | 1.80E-02 |
| PTPRS | 1.35 | 2.54 | 5.31E-04 | 2.07E-02 |
| CFHR5 | 0.20 | 1.15 | 6.28E-04 | 2.24E-02 |
| SERPINA10 | 0.23 | 1.18 | 7.52E-04 | 2.37E-02 |
| ITIH3 | 0.17 | 1.12 | 9.60E-04 | 2.81E-02 |
| PTPRD | 0.98 | 1.97 | 1.10E-03 | 3.12E-02 |
| PON3 | 0.39 | 1.31 | 1.15E-03 | 3.14E-02 |
| FGL1 | 1.26 | 2.39 | 1.26E-03 | 3.33E-02 |
| MMP2 | 0.26 | 1.20 | 1.44E-03 | 3.65E-02 |
| C1RL | 0.26 | 1.20 | 1.47E-03 | 3.65E-02 |
| SAA1 | 1.41 | 2.65 | 1.72E-03 | 4.02E-02 |
| REG1A | 1.38 | 2.60 | 1.77E-03 | 4.03E-02 |
| COL6A3 | 0.29 | 1.22 | 2.15E-03 | 4.66E-02 |
| B3GNT8 | 0.94 | 1.92 | 2.36E-03 | 4.71E-02 |

Differentially expressed proteins were identified by the following criteria: (1)｜log_2_ FC｜> 0.1375 ; and (2) the *p*-value after the FDR multiple test correction (*q* value) < 0.05 by Benjamini-Hochberg method. FC: fold change; SHBG: sex hormone-binding globulin; CD14: monocyte differentiation antigen CD14; TFRC: transferrin receptor protein 1; LBP: lipopolysaccharide-binding protein; C9: complement component C9; APOA4: apolipoprotein A-IV; LRG1: leucine-rich alpha-2-glycoprotein; QSOX1: sulfhydryl oxidase 1; ADAMDEC1: disintegrin and metalloprotease-like decysin-1; CFHR3: complement factor H-related protein 3; PTPRS: receptor-type tyrosine-protein phosphatase S; CFHR5: complement factor H-related protein 5; SERPINA10: protein Z-dependent protease inhibitor; ITIH3: inter-alpha-trypsin inhibitor heavy chain H3; PTPRD: receptor-type tyrosine-protein phosphatase delta; PON3: serum paraoxonase/lactonase 3; FGL1: fibrinogen-like protein 1; MMP2: 72 kDa type IV collagenase; C1RL: complement C1r subcomponent-like protein; SAA1: serum amyloid A-1 protein; REG1A: lithostathine-1-alpha; COL6A3: collagen alpha-3(VI) chain; B3GNT8: UDP-GlcNAc:betaGal beta-1,3-N-acetylglucosaminyltransferase 8.
