# Supplementary material for: Effect of dapagliflozin on proteomics and metabolomics of serum from patients with type 2 diabetes
Source: Diabetol Metab Syndr. 2023 Dec 4;15:251. doi: 10.1186/s13098-023-01229-0 (PMC10694884; doi:10.1186/s13098-023-01229-0)
Supplement: Supplementary file 9 — Additional file 9: Table S2. Decreased proteins in the T2D patients after dapagliflozin treatment. [file 13098_2023_1229_MOESM9_ESM.docx]

Additional file 9: Table S2. Decreased proteins in the T2D patients after dapagliflozin treatment

| Protein | log2FC | *FC* | *p value* | *q value* |
| --- | --- | --- | --- | --- |
| ALDOB | -1.31 | 0.40 | 2.88E-09 | 2.36E-06 |
| C3 | -0.17 | 0.89 | 4.53E-06 | 1.24E-03 |
| FN1 | -0.35 | 0.79 | 7.78E-06 | 1.53E-03 |
| AFM | -0.19 | 0.88 | 3.19E-05 | 3.27E-03 |
| GBE1 | -1.82 | 0.28 | 4.18E-05 | 3.80E-03 |
| ATRN | -0.17 | 0.89 | 9.12E-05 | 7.47E-03 |
| HPD | -1.80 | 0.29 | 1.12E-04 | 8.30E-03 |
| ACY1 | -1.17 | 0.44 | 1.88E-04 | 1.10E-02 |
| ALDH1A1 | -0.76 | 0.59 | 2.78E-04 | 1.43E-02 |
| ALDH9A1 | -1.04 | 0.49 | 6.56E-04 | 2.24E-02 |
| APOL1 | -0.23 | 0.85 | 6.87E-04 | 2.25E-02 |
| THBS4 | -0.30 | 0.81 | 9.09E-04 | 2.76E-02 |
| PSMA4 | -0.93 | 0.52 | 2.16E-03 | 4.66E-02 |
| FBP1 | -1.61 | 0.33 | 2.26E-03 | 4.71E-02 |
| DDT | -1.67 | 0.32 | 2.32E-03 | 4.71E-02 |

Differentially expressed proteins were identified by the following criteria: (1)｜log_2_ FC｜> 0.1375 ; and (2) the *p*-value after the FDR multiple test correction (*q* value) < 0.05 by Benjamini-Hochberg method. FC: fold change; ALDOB: fructose-bisphosphate aldolase B; C3: Complement component C3; FN1: fibronectin; AFM: afamin; GBE1: 1,4-alpha-glucan-branching enzyme; ATRN: attractin; HPD: 4-hydroxyphenylpyruvate dioxygenase; ACY1: aminoacylase-1; ALDH1A1: retinal dehydrogenase 1; ALDH9A1: 4-trimethylaminobutyraldehyde dehydrogenase; APOL1: apolipoprotein L1; THBS4: thrombospondin-4; PSMA4: proteasome subunit alpha type-4; FBP1: fructose-1,6-bisphosphatase 1; DDT: D-dopachrome decarboxylase.
