# Supplementary material for: Effect of dapagliflozin on proteomics and metabolomics of serum from patients with type 2 diabetes
Source: Diabetol Metab Syndr. 2023 Dec 4;15:251. doi: 10.1186/s13098-023-01229-0 (PMC10694884; doi:10.1186/s13098-023-01229-0)
Supplement: Supplementary file 12 — Additional file 12: Table S5. Increased metabolites in the T2D patients after dapagliflozin treatment. [file 13098_2023_1229_MOESM12_ESM.docx]

Additional file 12: Table S5. Increased metabolites in the T2D patients after dapagliflozin treatment

| **metabolite** | **log2FC** | **FC** | ***p value*** | ***q value*** |
| --- | --- | --- | --- | --- |
| Pyrogallol-1-O-sulphate | 0.28 | 1.21 | 1.00E-06 | 6.41E-05 |
| **Isobutyryl-L-carnitine** | 0.53 | 1.45 | 2.35E-04 | 9.17E-03 |
| Asp-Leu | 0.97 | 1.97 | 2.93E-04 | 1.03E-02 |
| Decenoylcarnitine | 0.43 | 1.35 | 2.86E-04 | 1.03E-02 |
| Asp-Ile | 1.17 | 2.24 | 4.92E-04 | 1.51E-02 |
| Taurine | 0.33 | 1.25 | 4.91E-04 | 1.51E-02 |
| Asp-Phe | 1.47 | 2.78 | 7.97E-04 | 2.16E-02 |
| Hippuric acid | 0.59 | 1.50 | 7.86E-04 | 2.16E-02 |
| **Arachidyl carnitine** | 0.31 | 1.24 | 1.24E-03 | 2.98E-02 |
| Sphinganine 1-phosphate | 0.16 | 1.12 | 1.31E-03 | 2.98E-02 |
| Octadecanamide | 0.14 | 1.10 | 1.45E-03 | 3.19E-02 |
| **LysoPE(18:0p/0:0)** | 0.29 | 1.23 | 1.85E-03 | 3.48E-02 |
| Pyrocatechol sulfate | 0.15 | 1.11 | 1.88E-03 | 3.48E-02 |
| p-Cresol glucuronide | 0.59 | 1.50 | 2.38E-03 | 4.18E-02 |
| O-Methoxycatechol-O-sulphate | 0.20 | 1.15 | 2.43E-03 | 4.18E-02 |
| Citrulline | 0.14 | 1.10 | 2.78E-03 | 4.66E-02 |
| Imidazolelactic acid | 0.21 | 1.16 | 3.05E-03 | 4.73E-02 |

Differentially expressed proteins were identified by the following criteria: (1)｜log_2_ FC｜> 0.1375 ; and (2) the *p*-value after the FDR multiple test correction (*q* value) < 0.05 by Benjamini-Hochberg method. FC: fold change; Asp-Leu: aspartyl-leucine; Asp-Ile: aspartyl-isoleucine; Asp-Phe: aspartylphenylalanine.
