# Supplementary material for: Effect of dapagliflozin on proteomics and metabolomics of serum from patients with type 2 diabetes
Source: Diabetol Metab Syndr. 2023 Dec 4;15:251. doi: 10.1186/s13098-023-01229-0 (PMC10694884; doi:10.1186/s13098-023-01229-0)
Supplement: Supplementary file 13 — Additional file 13: Table S6. Decreased metabolites in the T2D patients after dapagliflozin treatment. [file 13098_2023_1229_MOESM13_ESM.docx]

Additional file 13: Table S6. Decreased metabolites in the T2D patients after dapagliflozin treatment

| **metabolite** | **log2FC** | **FC** | ***p value*** | ***q value*** |
| --- | --- | --- | --- | --- |
| 1,5-Anhydrosorbitol | -1.20 | 0.44 | 1.68E-09 | 6.90E-07 |
| Gluconolactone | -0.50 | 0.71 | 1.96E-09 | 6.90E-07 |
| Ribose | -0.41 | 0.75 | 8.95E-09 | 1.58E-06 |
| 3,4-Dihydroxybutyric acid | -0.36 | 0.78 | 8.81E-09 | 1.58E-06 |
| 3-Methylglutaconic acid | -0.43 | 0.74 | 1.31E-08 | 1.84E-06 |
| Methylsuccinic acid | -0.45 | 0.73 | 2.00E-08 | 2.16E-06 |
| 3-Hydroxymethylglutaric acid | -0.40 | 0.76 | 2.92E-08 | 2.29E-06 |
| Hexose | -0.38 | 0.77 | 2.64E-08 | 2.29E-06 |
| Glutaric acid | -0.44 | 0.74 | 4.22E-08 | 2.97E-06 |
| Suberic acid | -0.40 | 0.76 | 1.40E-05 | 8.19E-04 |
| Homo-L-Arg | -0.37 | 0.77 | 8.41E-05 | 4.23E-03 |
| Xanthine | -0.52 | 0.70 | 9.32E-05 | 4.38E-03 |
| Methyl beta-D-glucopyranoside | -0.57 | 0.67 | 1.22E-04 | 5.35E-03 |
| LysoPC (16:1/0:0) | -0.28 | 0.82 | 4.83E-04 | 1.51E-02 |
| Hydroxyphenyllactic acid | -0.26 | 0.83 | 6.07E-04 | 1.78E-02 |
| Succinic acid | -0.17 | 0.89 | 1.12E-03 | 2.93E-02 |
| Hematoporphyrin | -0.76 | 0.59 | 1.52E-03 | 3.20E-02 |
| Uric acid | -0.21 | 0.86 | 1.54E-03 | 3.20E-02 |
| LysoPC (14:0/0:0) | -0.31 | 0.80 | 3.15E-03 | 4.73E-02 |

Differentially expressed proteins were identified by the following criteria: (1)｜log_2_ FC｜> 0.1375 ; and (2) the *p*-value after the FDR multiple test correction (*q* value) < 0.05 by Benjamini-Hochberg method. FC: fold change; Homo-L-Arg: L-homoarginine.
